# Supplementary material for: Factors associated with Facebook addiction among university students amid the COVID-19 pandemic: Findings from an online cross-sectional survey
Source: PLoS One. 2022 Aug 25;17(8):e0272905. doi: 10.1371/journal.pone.0272905 (PMC9409578; doi:10.1371/journal.pone.0272905)
Supplement: Attachment — Submitted filename: PONE-D-22-08770_reviewer comments.pdf [file pone.0272905.s002.pdf]

PLOS ONE

Factors Associated with Facebook Addiction among Bangladeshi University Students amid the COVID-19 Pandemic: Findings from an Online Cross-Sectional Survey

--Manuscript Draft--

**Manuscript Number:**	PONE-D-22-08770
**Article Type:**	Research Article
**Full Title:**	Factors Associated with Facebook Addiction among Bangladeshi University Students amid the COVID-19 Pandemic: Findings from an Online Cross-Sectional Survey
**Short Title:**	Facebook Addiction among Bangladeshi University Students
**Corresponding Author:**	Rezaul Karim Ripon<br>Jahangirnagar University<br>Dhaka, BANGLADESH
**Keywords:**	Facebook addiction; Bangladeshi university student; COVID-19
**Abstract:**	Background<br><br>There is a severe lack of research evidence regarding the Facebook addiction behavior among Bangladeshi university students during the ongoing COVID-19 pandemic.<br><br>Aim<br><br>The aim of this study was to determine factors associated with Facebook addiction among Bangladeshi university students.<br><br>Methods<br><br>A cross-sectional online survey was conducted among 2,161 Bangladeshi students during the COVID-19 pandemic.<br><br>Results<br><br>Female respondents and those whose family monthly income was <25,000 BDT were more addicted to Facebook than others respondent. Respondents who lost a family member or a relative to COVID-19, engaged in physical activities (exercise) during the pandemic, used Facebook for work purposes, or used Facebook to relieve daily stress were more addicted to Facebook.<br><br>Conclusion<br><br>Overuse of social media is problematic as it can trigger several mental health symptoms, especially among students. . Adequate and effective interventions are required to educate students about the dangers of Facebook addiction and to provide alternative, healthy options.
**Order of Authors:**	Rezaul Karim Ripon
	Abdullah Al Zubayer
	Quazi Maksudur Rahman
	Abid Hasan Khan
	Arifur Rahaman
	M Tasdik Hasan
	Md. Rifat Al Mazid Bhuiyan
	Md. Kamrul Ahsan Khan
	Md Ashraf Uddin Chowdhury

Powered by Editorial Manager® and ProduXion Manager® from Aries Systems Corporation

	Md Zakir Hossain
**Additional Information:**	
**Question**	**Response**
**Financial Disclosure**<br><br>Enter a financial disclosure statement that describes the sources of funding for the work included in this submission. Review the submission guidelines for detailed requirements. View published research articles from *PLOS ONE* for specific examples.<br><br>This statement is required for submission and **will appear in the published article** if the submission is accepted. Please make sure it is accurate.<br><br>**Unfunded studies**<br>Enter: *The author(s) received no specific funding for this work.*<br><br>**Funded studies**<br>Enter a statement with the following details:<br>• Initials of the authors who received each award<br>• Grant numbers awarded to each author<br>• The full name of each funder<br>• URL of each funder website<br>• Did the sponsors or funders play any role in the study design, data collection and analysis, decision to publish, or preparation of the manuscript?<br>• **NO** - Include this sentence at the end of your statement: *The funders had no role in study design, data collection and analysis, decision to publish, or preparation of the manuscript.*<br>• **YES** - Specify the role(s) played.<br><br>* typeset	no
**Competing Interests**<br><br>Use the instructions below to enter a competing interest statement for this submission. On behalf of all authors, disclose any competing interests that could be perceived to bias this work—acknowledging all financial support and any other relevant financial or non-financial competing interests.	NO

This statement is required for submission and **will appear in the published article** if the submission is accepted. Please make sure it is accurate and that any funding sources listed in your Funding Information later in the submission form are also declared in your Financial Disclosure statement.<br><br>View published research articles from *PLOS ONE* for specific examples.<br><br>**NO authors have competing interests**<br><br>Enter: *The authors have declared that no competing interests exist.*<br><br>**Authors with competing interests**<br><br>Enter competing interest details beginning with this statement:<br><br>*I have read the journal's policy and the authors of this manuscript have the following competing interests: [insert competing interests here]*<br><br>* typeset	
**Ethics Statement**<br><br>Enter an ethics statement for this submission. This statement is required if the study involved:<br><br>• Human participants<br>• Human specimens or tissue<br>• Vertebrate animals or cephalopods<br>• Vertebrate embryos or tissues<br>• Field research<br><br>Write "N/A" if the submission does not require an ethics statement.<br><br>General guidance is provided below. Consult the submission guidelines for detailed instructions. **Make sure that all**	The study ethically approved by Sheikh Sayera Khatun Medical College, Gopalgonj ethical committee (SSKMC/EC/2021/476(B))

Powered by Editorial Manager® and ProduXion Manager® from Aries Systems Corporation

**information entered here is included in the Methods section of the manuscript.**<br><br>**Format for specific study types**<br><br>**Human Subject Research (involving human participants and/or tissue)**<br>• Give the name of the institutional review board or ethics committee that approved the study<br>• Include the approval number and/or a statement indicating approval of this research<br>• Indicate the form of consent obtained (written/oral) or the reason that consent was not obtained (e.g. the data were analyzed anonymously)<br><br>**Animal Research (involving vertebrate animals, embryos or tissues)**<br>• Provide the name of the Institutional Animal Care and Use Committee (IACUC) or other relevant ethics board that reviewed the study protocol, and indicate whether they approved this research or granted a formal waiver of ethical approval<br>• Include an approval number if one was obtained<br>• If the study involved *non-human primates*, add *additional details* about animal welfare and steps taken to ameliorate suffering<br>• If anesthesia, euthanasia, or any kind of animal sacrifice is part of the study, include briefly which substances and/or methods were applied<br><br>**Field Research**<br><br>Include the following details if this study involves the collection of plant, animal, or other materials from a natural setting:<br>• Field permit number<br>• Name of the institution or relevant body that granted permission	
**Data Availability**<br><br>Authors are required to make all data underlying the findings described fully available, without restriction, and from the time of publication. PLOS allows rare exceptions to address legal and ethical	Yes - all data are fully available without restriction

concerns. See the PLOS Data Policy and FAQ for detailed information.<br><br>A Data Availability Statement describing where the data can be found is required at submission. Your answers to this question constitute the Data Availability Statement and **will be published in the article**, if accepted.<br><br>**Important:** Stating 'data available on request from the author' is not sufficient. If your data are only available upon request, select 'No' for the first question and explain your exceptional situation in the text box.<br><br>Do the authors confirm that all data underlying the findings described in their manuscript are fully available without restriction?	
**Describe where the data may be found in full sentences. If you are copying our sample text, replace any instances of XXX with the appropriate details.**<br><br>• If the data are **held or will be held in a public repository**, include URLs, accession numbers or DOIs. If this information will only be available after acceptance, indicate this by ticking the box below. For example: *All XXX files are available from the XXX database (accession number(s) XXX, XXX.*).<br>• If the data are all contained **within the manuscript and/or Supporting Information files**, enter the following: *All relevant data are within the manuscript and its Supporting Information files.*<br>• If neither of these applies but you are able to provide **details of access elsewhere**, with or without limitations, please do so. For example:<br><br>*Data cannot be shared publicly because of [XXX]. Data are available from the XXX Institutional Data Access / Ethics Committee (contact via XXX) for researchers who meet the criteria for access to confidential data.*	NO

The data underlying the results presented in the study are available from (include the name of the third party and contact information or URL).<br>• This text is appropriate if the data are owned by a third party and authors do not have permission to share the data.<br><br>* typeset	
Additional data availability information:	Tick here if the URLs/accession numbers/DOIs will be available only after acceptance of the manuscript for publication so that we can ensure their inclusion before publication.; Tick here if your circumstances are not covered by the questions above and you need the journal’s help to make your data available.

Powered by Editorial Manager® and ProduXion Manager® from Aries Systems Corporation

Click here to access/download;Manuscript;Facebook addiction draft manuscript_MTH29Nov21.docx

Factors Associated with Facebook Addiction among Bangladeshi University Students amid the COVID-19 Pandemic: Findings from an Online Cross-Sectional Survey

Rezaul Karim Ripon[1], Abdullah Al Zubayer[2], Quazi Maksudur Rahman[1], Abid Hasan Khan[1], Arifur Rahaman[3], M Tasdik Hasan [4,5], Md. Rifat Al Mazid Bhuiyan [6], Md. Kamrul Ahsan Khan[7], Md Ashraf Uddin Chowdhury [7], Md Zakir Hossain [7]

[1]Department of Public Health and Informatics, Jahangirnagar University, Savar, Dhaka-1342, Bangladesh

[2]Department of Sociology, University of Barishal, Barishal, Bangladesh

[3]Department of Sociology, University of Dhaka

[4]Jeeon Bangladesh Ltd., Dhaka,

[5] Bangladesh Public Health Foundation, Bangladesh, Dhaka, Bangladesh

[6]Dhaka Community Medical College, Mogbazar, Dhaka, Bangladesh

[7]Sheikh Sayera Khatun Medical College, Gopalgonj

*Corresponding author

Rezaul Karim Ripon

Department of Public Health and Informatics

Jahangirnagar University, Savar, Dhaka, Bangladesh

Email: riponrezaul5@gmail.com

Mobile: +8801535480143

Abstract:

Background: There is a severe lack of research evidence regarding the Facebook addiction behavior among Bangladeshi university students during the ongoing COVID-19 pandemic.

Aim: The aim of this study was to determine factors associated with Facebook addiction among Bangladeshi university students.

Methods: A cross-sectional online survey was conducted among 2,161 Bangladeshi students during the COVID-19 pandemic.

Results: Female respondents and those whose family monthly income was <25,000 BDT were more addicted to Facebook than others respondent. Respondents who lost a family member or a relative to COVID-19, engaged in physical activities (exercise) during the pandemic, used Facebook for work purposes, or used Facebook to relieve daily stress were more addicted to Facebook.

Conclusion: Overuse of social media is problematic as it can trigger several mental health symptoms, especially among students. . Adequate and effective interventions are required to educate students about the dangers of Facebook addiction and to provide alternative, healthy options.

Keywords: Facebook addiction, Bangladeshi university student, COVID-19;

Introduction:

Our lives have been simplified by the growth of technology-mediated help in delivering better communication services, but behavioral addictions such as internet addiction and social media addiction have become common as a result (Kuss and Griffiths, 2011; Rajesh and Rangaiah, 2020). Among many social networking sites, Facebook has grown in popularity as a result of the emergence of social networking sites, and it has been accompanied by a growing body of research. Facebook currently has 2.895 billion monthly active users (MAUs) and almost 125.46 million users in Bangladesh (BTRC, 2021). Young people tend to be the primary users of Facebook and other social networking sites, and for them, excessive usage of Facebook might be addictive (Koc and Gulyagci 2013; Przepiorka and Blachnio 2016). New hand-held gadgets, such as smartphones, tablets, and laptop computers, are increasing Internet access and portability, making it feasible to work online and enjoy leisure activities while on the go (Berner et al. 2018).

Facebook addiction (FA) can be classified as one of the aspects of Internet addiction (IA) (Mamun and Griffiths, 2019). Facebook addiction is a term coined by researchers that is applied to individuals who engage in excessive, compulsive Facebook use for the purposes of mood alteration, with negative personal outcomes (Ryan et al. 2014). Facebook addiction refers to dependency on Facebook caused by excessive use, which disrupts daily activities (Ahmed and Hossain, 2018). Problematic Facebook use has been

defined as Facebook use that creates problems in users' lives, such as psychological, emotional, social, school, or work difficulties (Marino et al., 2018).

The six criteria of addiction (salience, mood modification, tolerance, withdrawal, conflict, and relapse) or similar factors based on the definition of gambling addiction (e.g., withdrawal, interpersonal problems due to Facebook use, time management, and performance problems) are used to define Facebook addiction (Andreassen et al., 2012; Griffiths, 2005). Reasons and motivations behind Facebook's popularity include being able to easily access updated features which facilitate communicating with friends, meeting others based on shared interests, sharing pictures and videos, blogging, dating, and even gaming (Griffiths, 2015; Kuss & Griffiths, 2011; Ryan et al., 2014). Griffiths (2012), on the other hand, stated that people's activities on Facebook might include things like gaming and gambling, in addition to social networking. Most of Facebook users find comfort, life satisfaction, happiness, and social support while using this platform, although, others are connected with it as a means of escapism (Brailovskaia et al., 2018; Ryan et al., 2014). Facebook addiction is associated with many negative psychological traits (Hong et al., 2014). Ordinary Facebook users differ statistically in self-esteem and life satisfaction from both addicted and intensive users (Agata et al., 2016).

There are numerous factors that have been reported in relation to social media use more generally, and Facebook use more specifically. Sociodemographic factors such as age, gender, relationship status, and occupational/educational status can all play important roles in determining patterns of Facebook use (Sayeed et al., 2020; Soron and Tarafder, 2015; Lee and Cho, 2011). Predictors of problematic Facebook use have included a wide range of activities and factors, including less engagement in physical activity (Brailovskaia et al., 2020; Brailovskaia, Teismann and Margraf, 2018; Paschoal et al., 2020), loneliness (Shettar et al., 2017; Rajesh and Rangaiah, 2020), poor sleep (Ho, 2021; Ho et al., 2021; Wolniczak, 2013), and relationship disengagement (Abbasi, 2018). Extraversion, narcissism, high degrees of neuroticism, and low levels of self-esteem all have a strong correlation with excessive Facebook use (Mehdizadeh, 2010). Shyness, loneliness, and negative affect were found to be positively connected with FA (Satici, 2019). Besides, failure in love, a history

of domestic violence, a stressful life event, and sleep disturbances were all identified as risk factors for Facebook addiction in a previous study in Bangladesh (Sayeed et al., 2020).

SARS-CoV-2, or Covid-19, has emerged as the most serious public health threat of the contemporary age. No other events in the last few decades have had half the devastating effect as the COVID-19 pandemic is having now. More than 5 million individuals have died as a result of this infectious virus, which has infected about 200 million people. The coronavirus spread quickly across the world due to its infectious nature, resulting in a large number of deaths. Covid-19 brought about many changes in daily lives and lifestyles around the globe (e.g., working from home, home quarantine, home-schooling, etc.) and many new norms as preventive measures (e.g., wearing masks, sanitation, social distancing, and vaccination). All these together formed a 'new normal' environment coping with the challenges of the COVID-19 pandemic while conforming to preventive measures (Corpuz, 2021). In many ways, COVID-19 had an impact on nearly every nation in economic, social, cultural, political, and other spheres. Addiction to social media sites increased during COVID-19 owing to quarantine, social distancing, and other health measures (Li et al., 2021; Boursier et al., 2020).

Several studies have been conducted on Smartphone and Internet addiction during the first wave of the COVID pandemic (Hosen et al., 2021; Jahan et al., 2021; Islam et al., 2021). The objectives of this paper were to determine the factors associated with FA among university students, as well as make possible significant recommendations for taking appropriate preventive measures during the second wave of COVID-19, while undergoing a long-term home stay unlike any other. To assess the Facebook addiction behavior among the students, the study used the Bergen Facebook Addiction Scale (BFAS). The Bergen scale has been validated by several studies in Bangladesh (Ahmed & Hossain, 2018; Mamun & Griffiths, 2019).

Methods

Participants and procedure of data collection

During a subsequent wave of the COVID-19 pandemic in early 2021, this online cross-sectional survey was conducted to assess addiction to Facebook and its associated factors in Bangladesh among university students. The survey took place from June 2021 to September 2021. Participants were recruited through social media as per convenience sampling by using Google Form. Inclusion criteria included: being a Bangladeshi university student (age at least 18 years or older), having internet access, and residing in Bangladesh during the study. Those who are below 18 years old and not willing to participate were excluded. A sufficient number of research assistants were recruited to get a high response rate in the survey. On the first page of the electronic survey, the participants gave their written consent by using the yes/ no option. The survey took approximately 15/20 minutes to complete. The participation in this survey was on a volunteer basis and was anonymous. The study ethically approved by Sheikh Sayera Khatun Medical College, Gopalgonj ethical committee (SSKMC/EC/2021/476(B)). Initially, there were 2,170 responses, and after removing incomplete data, there were a total of 2161 responses.

Measures:

The aim of this study was to identify the prevalence and factors associated with Facebook addiction among university students amid the COVID-19 pandemic. The semi-structured Google form had several sections: a) socio-demographic information alongside a participant consent form. b) COVID-19-related information; and c) the Burgen Facebook Addiction Scale (BFAC) (Andreassen & Brunborg, 2012).

Socio-demographic measures:

Information on socio-demographic variables including: age (15 to 20 years, 21 to 25 years, 26 to 30 years, 30 years or more), gender(Female, Male), educational background (Science, Business studies, Social science, Arts, Humanitarian studies, others), family monthly

income(<25,000 BDT, 25,000-50,000 BDT, >50,000 BDT), marital status (bachelor, married, others), type of family(nuclear family, combined Family), current location(rural, urban).

Addiction in Facebook:

A validated Facebook addiction scale (Burgen Facebook Addiction BFAC) (Andreassen & Brunborg, 2012), is a six-item self-reported scale that is a brief and effective psychometric instrument for assessing at-risk social media addiction on the internet, used to asses addiction on Facebook. It is a 5 point Likert scale with (1) very rarely, (2) rarely, (3) sometimes, (4) often, and (5) very often. The overall score ranged from 6 to 30, with higher scores reflecting a greater addiction to Facebook (Andreassen et al. 2012). Using a score of 3 or more in response to four of the six items is an indicator of addiction (Andreassen et al.2012).

COVID-19 related information

COVID-19 related information consists of twelve questions. The Cronbach alpha for these questions was 0.864. The question responses were yes or no. *Are you separated from your family because of COVID-19? Were you in quarantine during this pandemic? Do you feel detached from your friends/peers in this pandemic? Are you dealing with relationship issues like family feuds or breakups?Do you feel lonely? Is your movement restricted because of a lockdown? Do you feel depressed about this pandemic? , Have you lost any family members or relatives to COVID-19? , andHave you thought about suicide at least once in this pandemic situation? , andAre you involved in any physical activities (exercise)? , How much time (hours) on average do you spend on Facebook in a day? , andWhat is your reason for using Facebook? (a. Work; b. Education; c. Upkeep of social networks.)d. Relief from day-to-day stress e. Passage of time f. Absence of a specific reason).*

Statistical analysis

Descriptive and inferential statistics (such as frequencies, percentages, means, and standard deviations) were computed. Inferential statistics included using t-tests or one-way Analysis of Variance (ANOVA) to determine mean differences in Facebook addiction and COVID-19 related questions in relation to sociodemographic variables. A well fitted regression model was used to assess the correlation between Facebook addiction and COVID-19 relationship-related questions. All analyses were carried out with a p-value less than 0.05 using the R programming language.

Results

Demographic analysis

A total 2,161 respondents were included in this study. Among them Age: 15 to 20 years (265, 12.3%), 21 to 25 years(1724, 79.8%), 26 to 30 years (146,6.8%), 30 years or more(26,1.2%); Gender: Female(1130, 52.3%), Male(1031,47.7%); Educational background: Science (1142,52.8%), Business studies(307, 14.2%), Social science(459, 21.2%), Arts (134,6.2%), Humanitarian studies(119,5.5%), others(0%); Family Monthly Income: <25,000 BDT (783,36.2%), 25,000-50,000 BDT(904,41.8%), >50,000 BDT (474,21.9%); Marital status: Bachelor(1930,89.3%), Married(224,10.4%), Others(7,.3%); Type of family: Nuclear family(1821, 84.3%), Combined Family (340,15.7%); Current location : Rural (.03,27.9%), Urban (1558,72.1%) [Table: 1].

Association of the demographic variable and respond with addiction in Facebook.

The overall Facebook addiction among university students in Bangladesh was 28.3% (n= 612). Among the respondent of bachelor marital status were 89.31 %(n=548,), Nuclear family 84.27% (n=509), Urban responded 72.10% (n=438), Age 21 to 25 years 79.78% (n=492), Female responded 52.29% (n=348), Educational background Science 52.85 %(n=302), Family Monthly Income 25,000-50,000 BDT 41.83% (n=254). Female respondents were 1.3 times more addicted to Facebook than male [OR (95% of CI, p value):1.293(1.071-1.561), .008]. Family Monthly Income <25,000 BDT were 1.4 times more addicted to Facebook than those monthly

income were >50,000 BDT [OR (95% of CI, p value): 1.430(1.103-1.853), .007]. And they remain significant after adjusting all other variables [Table: 2].

Association of the COVID-19 related question and positive response with addiction in Facebook.

Because of COVID-19 3.24%(n=70) separated from their family , 13.84%(n=299) were in quarantine in this pandemic, 19.81%(n=428) feel detached from their friends/peers in this pandemic, 16.66%(n=360) were dealing with relationship problem such as family conflicts or break ups in this pandemic. 19.11 %(n=413) feel lonely in this pandemic. 24.90%(n=538) movement were restricted because of lockdown, 24.20%(n=523) feel depressed in this pandemic, 7.08%(n=153) lost any family member or relatives to COVID-19, 9.76%(n=211) thought about suicide at least once in this pandemic situation, 8.98%(n=194) involved in any physical activities (exercise), during pandemic on average you spend on Facebook in a day: 0 to 5 hours 10.74%(n=232), 6 to 10 hours 10.18%(n=220), 11 hours or more 7.40%(n=160); reason for using Facebook: Work 1.48%(n=32), Maintain social Network 7.91%(n=171) , Relief daily stress 10.27%(n=222), Time passing 10.74%(n=232), No-reason 18.05%(n=390). Those feeling detached from your friends/peers in this pandemic were 2.5 times more addicted to Facebook than those who are not. 2.530(2.072-3.088), .0001], this variable also remains significant after adjusting all other variables. Responded who lost any family member or relatives to COVID-19 were 1.3 time higher addicted in Facebook than those are not [OR (95% of CI), p value: 1.322(1.059-1.649), .013] and this variable didn't significant after adjusting all other variables. And those involved in any physical activities (exercise) in this pandemic were 1.5 times more addicted in Facebook than those are not [OR(95% of CI), p value : 1.497(1.228-1.825), .0001] and this variable also didn't significant after adjusting all other variables. Those responded use Facebook for Work purpose were 1.2 times more addicted in Facebook than those are not [OR (95% of CI), p value: 1.19(1.24-3.1), .042], responded use Facebook for Relief daily stress purpose were 1.5 times more addicted in Facebook than those are not [OR (95% of CI), p value: 1.536(1.214-2.246), .003] and these variable remain significant after adjusting all other variables [Table: 3].

4. Discussion:

This study explored several factors associated with Facebook addiction among Bangladeshi university students. In our study, it was highlighted that females were more addicted to Facebook than their male counterparts. Prior studies found that the COVID-19 outbreak has severely constrained people's daily lives and increased their use of social media (J. Brailovskaia & Margraf, 2021) and about forty percent of women reported problematic social media (Facebook) use (Sediri et al., 2020), along with in the model of Facebook addiction, the gender of the female would be significant (Cudo et al., 2020). Besides, it was seen that those whose monthly family income was less than twenty five thousand (BDT) were more addicted to Facebook than others. Previous studies disclosed that, level of income does not show significant influence on the risk of Facebook addiction (Jafarkarimi et al., 2016). This kind of dissimilarity appears to be due to geographical location and time variation (pandemic situation). Our study found that students who felt disconnected from friends or peers during the COVID-19 pandemic had higher Facebook addiction, whereas a previous study found that physical separation encouraged people to use the Internet for virtually all daily tasks during the pandemic (Siste et al., 2020).In our study, it was also highlighted that students who lost any family member or relative to COVID-19 were more addicted to Facebook, which may be the reason for suffering from a stressful life event due to the loss of beloved members. This is similar to prior studies in Bangladesh that found having a stressful life event was responsible for Facebook addiction (Sayeed et al., 2020). This study revealed that those involved in any physical activities in this pandemic had higher Facebook addiction. A prior study also showed that problematic smartphone use is linked to physical inactivity during this COVID-19 outbreak (Hosen et al., 2021) and that these trends in smartphone use may increase the use of Facebook, which turns into addiction. But the inconsistency between these two findings may vary due to the diverse perceptions among university students. However, our study reported that students using Facebook for work purposes (e.g. online marketing) were more addicted to Facebook, whereas a previous study showed that broadcasting behavior on Facebook positively predicts Facebook addiction (Xie & Karan, 2019). Furthermore, this study discovered that students who used Facebook to relieve daily stress were more likely to become Facebook addicts. Previous studies considered that time spent using Facebook is one of the main predictors of Facebook addiction

(Cudo et al., 2020), and daily stress was linked to the amount of time spent on Facebook and the likelihood of becoming addicted to it. The relationship between daily stress and Facebook use intensity was negatively moderated by perceived offline social support, implying that people who got low levels of support offline were more inclined to increase their Facebook use when they were under stress (Julia Brailovskaia et al., 2019). In addition, governmental and non-governmental organizations should come forward to take appropriate strategies to overcome this vulnerable condition, such as Facebook addiction, for the well-being of university students to protect the future leaders of the nation.

4.1. Limitations of the study:

There were a few drawbacks to this study. To begin with, the study's self-reported data is prone to reporting bias, and respondents from lower socioeconomic classes who do not have access to the internet or Wi-Fi were not included in the study. Second, selection bias is a limitation of the convenience sampling technique. Finally, due to the study's cross-sectional design, it was difficult to investigate any possible causality. Further research into these topics should be done using a mixed-methods approach with large-scale studies. Despite the study's limitations, we feel it provides vital evidence on university students' Facebook addiction after a year of living with the COVID-19 outbreak.

Abbreviations:

Not applicable

Declaration:

Ethics approval and consent to participate: SSKMC/EC/2021/476(B)

Consent for publication: No

Availability of data and material: On request

Competing interests: No

Funding: Self-funded

Authors' contributions: RKR data analysis, first draft, review; AAZ project planner, review the draft, QMR project planner, writing first draft **,** AHK project planner , AR writing first draft, MTH review the draft , MRAMB project planner , MKAK project planner [7], MAUC project planner , M ZH project planner

Acknowledgements: The research assistants who help to collect the data.

References

Bangladesh Telecommunication Regulatory Commission. *Internet Subscribers in Bangladesh*, October. (2021). Available online at: http://www.btrc.gov.bd/content/internet-subscribers-bangladesh-august-2021

Bener, A., Yildirim, E., Torun, P., Çatan, F., Bolat, E., Alıç, S., & Griffiths, M. D. (2019). Internet addiction, fatigue, and sleep problems among adolescent students: A large-scale study. *International Journal of Mental Health and Addiction*, *17*(4), 959-969.

Boursier, V., Gioia, F., Musetti, A., & Schimmenti, A. (2020). Facing loneliness and anxiety during the COVID-19 isolation: the role of excessive social media use in a sample of Italian adults. *Frontiers in psychiatry*.

Brailovskaia, J., Ströse, F., Schillack, H., & Margraf, J. (2020). Less Facebook use–More well-being and a healthier lifestyle? An experimental intervention study. *Computers in Human Behavior*, 108, 106332.

Brailovskaia, J., Teismann, T., & Margraf, J. (2018). Physical activity mediates the association between daily stress and Facebook addiction disorder (FAD)–A longitudinal approach among German students. *Computers in Human Behavior*, 86, 199-204.

Griffiths, M. (2005). A 'components' model of addiction within a biopsychosocial framework. *Journal of Substance use*, *10*(4), 191-197.

Ho, T. T. Q. (2021). Facebook addiction and depression: Loneliness as a moderator and poor sleep quality as a mediator. *Telematics and Informatics*, 61, 101617.

Hosen, I., Al Mamun, F., Sikder, M. T., Abbasi, A. Z., Zou, L., Guo, T., & Mamun, M. A. (2021). Prevalence and associated factors of problematic smartphone use during the COVID-19 pandemic: a Bangladeshi study. *Risk Management and Healthcare Policy*, 14, 3797.

Jahan, I., Hosen, I., Al Mamun, F., Kaggwa, M. M., Griffiths, M. D., & Mamun, M. A. (2021). How has the COVID-19 pandemic impacted internet use behaviors and facilitated problematic internet use? A Bangladeshi study. *Psychology Research and Behavior Management*, 14, 1127.

Koc, M., & Gulyagci, S. (2013). Facebook addiction among Turkish college students: the role of psychological health, demographic, and usage characteristics. *Cyberpsychology, Behavior, and Social Networking*, 16(4), 279–284.

Kuss, D. J., & Griffiths, M. D. (2011). Online social networking and addiction—a review of the psychological literature. *International journal of environmental research and public health*, *8*(9), 3528-3552.

Li, Y. Y., Sun, Y., Meng, S. Q., Bao, Y. P., Cheng, J. L., Chang, X. W., ... & Shi, J. (2021). Internet Addiction Increases in the General Population During COVID- 19: Evidence From China. *The American Journal on Addictions*.

Mamun, M., & Griffiths, M. D. (2019). The association between Facebook addiction and depression: A pilot survey study among Bangladeshi students. *Psychiatry research*, *271*, 628–633. https://doi.org/10.1016/j.psychres.2018.12.039

Marino, C., Gini, G., Vieno, A., & Spada, M. M. (2018). The associations between problematic Facebook use, psychological distress and well-being among adolescents and young adults: A systematic review and meta-analysis. *Journal of Affective Disorders*, *226,* 274-281.

Przepiorka, A., & Blachnio, A. (2016). Time perspective in Internet and Facebook addiction. *Computers in Human Behavior*, 60,13–18

Saeed Abbasi, I. (2018). The link between romantic disengagement and Facebook addiction: Where does relationship commitment fit in?. *The american journal of family therapy*, 46(4), 375-389.

Schou Andreassen, C., & Pallesen, S. (2014). Social network site addiction-an overview. *Current pharmaceutical design*, *20*(25), 4053-4061.

Shettar, M., Karkal, R., Kakunje, A., Mendonsa, R. D., & Chandran, V. M. (2017). Facebook addiction and loneliness in the post-graduate students of a university in southern India. *International Journal of Social Psychiatry*, 63(4), 325-329.

Wolniczak, I., Cáceres-DelAguila, J. A., Palma-Ardiles, G., Arroyo, K. J., Solís-Visscher, R., Paredes-Yauri, S., & Bernabe-Ortiz, A. (2013). Association between Facebook dependence and poor sleep quality: a study in a sample of undergraduate students in Peru. *PloS one*, 8(3), e59087.

Brailovskaia, J., & Margraf, J. (2021). The relationship between burden caused by coronavirus (Covid-19), addictive social media use, sense of control and anxiety. *Computers in Human Behavior*, *119*(January), 106720. https://doi.org/10.1016/j.chb.2021.106720

Brailovskaia, Julia, Rohmann, E., Bierhoff, H. W., Schillack, H., & Margraf, J. (2019). The relationship between daily stress, social support and Facebook Addiction Disorder. *Psychiatry Research*, *276*(May), 167–174. https://doi.org/10.1016/j.psychres.2019.05.014

Cudo, A., Torój, M., Demczuk, M., & Francuz, P. (2020). Dysfunction of Self-Control in Facebook Addiction: Impulsivity Is the Key. *Psychiatric Quarterly*, *91*(1), 91–101. https://doi.org/10.1007/s11126-019-09683-8

Hosen, I., Al Mamun, F., Sikder, M. T., Abbasi, A. Z., Zou, L., Guo, T., & Mamun, M. A. (2021). Prevalence and associated factors of problematic smartphone use during the covid-19 pandemic: A bangladeshi study. *Risk Management and Healthcare Policy*, *14*, 3797–3805. https://doi.org/10.2147/RMHP.S325126

Jafarkarimi, H., Sim, A. T. H., Saadatdoost, R., & Hee, J. M. (2016). Facebook Addiction among Malaysian Students. *International Journal of Information and Education Technology*, *6*(6), 465–469. https://doi.org/10.7763/ijiet.2016.v6.733

Sayeed, A., Hassan, M. N., Rahman, M. H., El Hayek, S., Banna, M. H. Al, Mallick, T., Hasan, A. R., Meem, A. E., & Kundu, S. (2020). Facebook addiction associated with internet activity, depression and behavioral factors among university students of Bangladesh: A cross-sectional study. *Children and Youth Services Review*, *118*, 105424. https://doi.org/10.1016/J.CHILDYOUTH.2020.105424

Sediri, S., Zgurb, Y., Quanes, S., Ouali, U., Bourgou, S., Jomli, R., & Nacef, F. (2020). Women's mental health: acute impact of COVID-19 pandemic on domestic violence. *Archives of Women's Mental Health*, *23*, 749–756.

Siste, K., Hanafi, E., Sen, L. T., Christian, H., Adrian, Siswidiani, L. P., Limawan, A. P., Murtani, B. J., & Suwartono, C. (2020). The Impact of Physical Distancing and Associated Factors Towards Internet Addiction Among Adults in Indonesia During COVID-19 Pandemic: A Nationwide Web-Based Study. *Frontiers in Psychiatry*, *11*(September), 1–11. https://doi.org/10.3389/fpsyt.2020.580977

Xie, W., & Karan, K. (2019). Predicting Facebook addiction and state anxiety without Facebook by gender, trait anxiety, Facebook intensity, and different Facebook activities. *Journal of Behavioral Addictions*, *8*(1), 79–87. https://doi.org/10.1556/2006.8.2019.09

Table 1 Prevalence Demographic Information

Demographic Variables		Frequency (%)
Age	15 to 20 years	265(12.3%)
	21 to 25 years	1724(79.8%)
	26 to 30 years	146(6.8%)
	30 years or more	26(1.2%)
Gender	Female	1130(52.3%)
	Male	1031(47.7%)
Educational background	Science	1142(52.8%)
	Business studies	307(14.2%)
	Social science	459(21.2%)
	Arts	134(6.2%)
	Humanitarian studies	119(5.5%)
Family Monthly Income	<25,000 BDT	783(36.2%)
	25,000-50,000 BDT	904(41.8%)
	>50,000 BDT	474(21.9%)
Marital status	Bachelor	1930(89.3%)
	Married	224(10.4%)
	Others	7(.3%)
Type of family	Nuclear family	1821(84.3%)
	Combined Family	340(15.7%)
Current location	Rural	603(27.9%)
	Urban	1558(72.1%)
Addiction in Facebook	Yes	612(28.3%)

	No	1549(71.7%)

Table 2 Association of the demographic variable and respond with addicted in Facebook.

Demographic Variable		Addicted in Facebook		
		Frequency (%)	OR(95% C.I.), P value	aOR(95% C.I.), P value
Age	15 to 20 years	81(12.26%)	.832(.356-1.944), .670	.727(.297-1.777), .484
	21 to 25 years	492(79.78%)	.754(.334-1.704), .498	.677(.287-1.596), .372
	26 to 30 years	30(6.76%)	.489(.198-1.204), .120	.477(.189-1.200), .116
	30 years or more	9(1.20%)	1	1
Gender	Female	348(52.29%)	1.293(1.071-1.561), .008	1.323(1.087-1.610), .005
	Male	264(47.71%)	1	1
Educational background	Science	302(52.85%)	.766(.510-1.152), .200	.833(.548-1.265), .391
	Business studies	85(14.21%)	.816(.516-1.292), .386	.907(.568-1.447), .682
	Social science	147(21.24%)	1.004(.652-1.548), .984	1.078(.693-1.677), .738
	Arts	40(6.20%)	.907(.532-1.548), .720	.962(.560-1.650), .887
	Humanitarian studies	38(5.51%)	1	1
Family Monthly Income	<25,000 BDT	244(36.23%)	1.430(1.103-1.853), .007	1.455(1.107-1.913), .007
	25,000-50,000 BDT	254(41.83%)	1.234(.956-1.594), .107	1.260(.972-1.634), .081
	>50,000 BDT	114(21.93%)	1	1
Marital status	Bachelor	548(89.31%)	1.036(.761-1.411), .822	.873(.162-4.699), .874
	Married	62(10.37%)	.775	.780(.143-4.265), .775
	Others	2(0.32%)	1	1
Type of family	Nuclear family	509(84.27%)	.893(.693-1.150), .379	.895(.656-1.221), .482
	Combined Family	103(15.73%)	.873(.162-4.699), .874	1
Current location	Rural	174(27.90%)	1.037(.842-1.277), .731	.976(.779-1.222), .831
	Urban	438(72.10%)	1	1

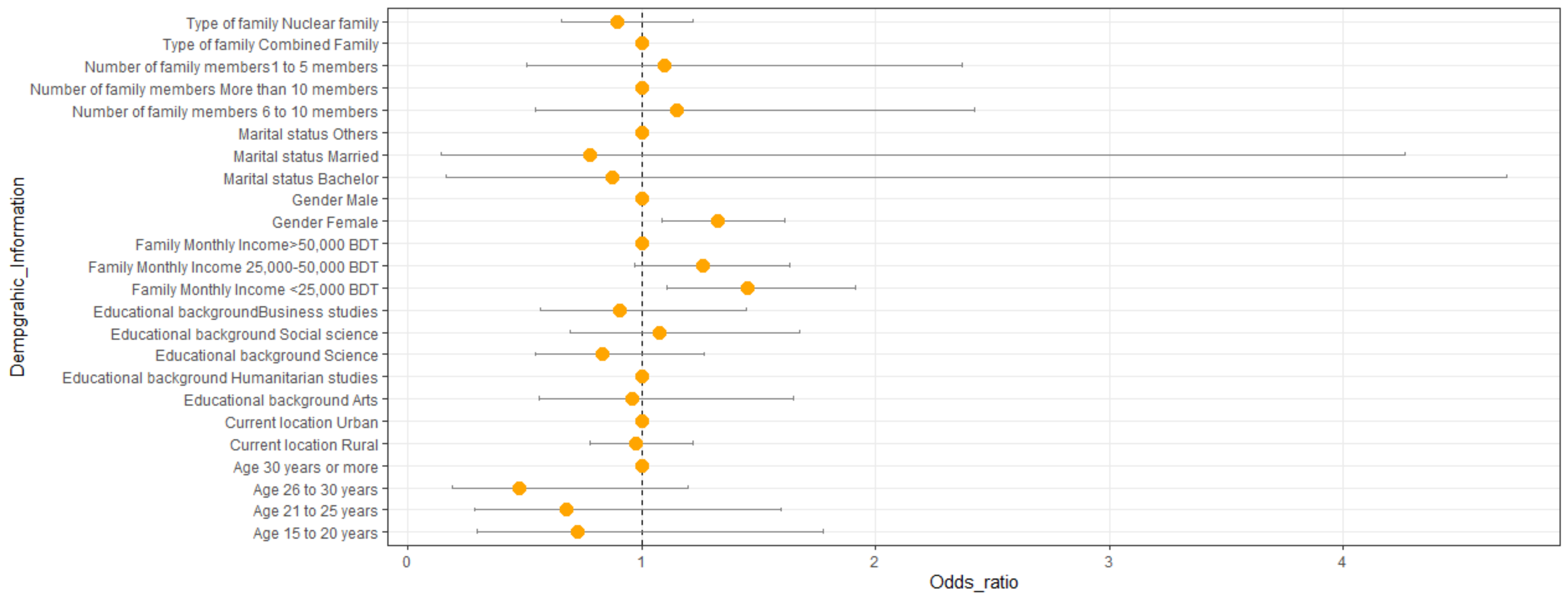
Type of family Nuclear family
Type of family Combined Family
Number of family members1 to 5 members
Number of family members More than 10 members
Number of family members 6 to 10 members
Marital status Others
Marital status Married
Marital status Bachelor
Gender Male
Gender Female
Family Monthly Income>50,000 BDT
Family Monthly Income 25,000-50,000 BDT
Family Monthly Income <25,000 BDT
Educational backgroundBusiness studies
Educational background Social science
Educational background Science
Educational background Humanitarian studies
Educational background Arts
Current location Urban
Current location Rural
Age 30 years or more
Age 26 to 30 years
Age 21 to 25 years
Age 15 to 20 years
Dempgrahic_Information
0
1
2
3
4
Odds_ratio

Table 3 Association of the COVID-19 related question and positive respond with addicted in Facebook.

COVID-19 related questions *		Addicted in Facebook		
		Frequency (%)	OR(95% C.I.), p value	aOR(95% C.I.), p value
Are you separated from your family because COVID-19?		70(3.24%)	948(.705-1.275), .725	1.139(.821-1.580), .437
Were you in quarantine in this pandemic?		299(13.84%)	.958(.794-1.155), .650	1.214(.984-1.498), .071
Do you feel detached from your friends/peers in this pandemic?		428(19.81%)	2.530(2.072-3.088), .0001	.645(.508-.819), .0001
Are you dealing with relationship problem such as family conflicts or break ups?		360(16.66%)	.435(.360-.527), .0001	.712(.570-.889), .003
Do you feel lonely?		413(19.11%)	.322(.264-.392), .0001	.604(.473-.770), .0001
Is your movement restricted because of lockdown?		538(24.90%)	.623(.473-.821), .001	.969(.702-1.337), 848
Do you feel depressed in this pandemic?		523(24.20%)	.374(.292-.480), .0001	.647(.484-.864)
Have you lost any family member or relatives to COVID-19?		153(7.08%)	1.322(1.059-1.649), .013	.863(.675-1.103), .238
Have your thought about suicide at least once in this pandemic situation?		211(9.76%)	.412(.334-.509), .0001	.809(.633-1.035), .091
Are you involved in any physical activities (exercise)?		194(8.98%)	1.497(1.228-1.825), .0001	1.2439(.998-1.547), .052
How much time (hours) on average you spend on Facebook in a day?	0 to 5 hours	232(10.74%)	.132(.112-.984), .005	.282(.212-.374), .0001
	6 to 10 hours	220(10.18%)	.214(.101-.954), .004	.647(.481-.872), .004
	11 hours or more	160(7.40%)	1	1
What is your reason for using Facebook?	Work	32(1.48%)	1.19(1.24-3.1), .042	1.619(1.044-2.5090, .031
	Maintain social_ Network	171(7.91%)	1.8(.765-2.05), .087	1.258(.985-1.605), .066

	Relief_ daily stress	222(10.27%)	1.536(1.214-2.246), .003	1.376(1.084-1.746), .009
	Time_ passing	232(10.74%)	.641(.123-.740), .06	.741(.583-.940), .014
	No-reason	390(18.05%)	.12(.10-.653), 0.32	.692(.555-.863), .05

- No used as a reference categories.
