# Supplementary material for: Factors associated with Facebook addiction among university students amid the COVID-19 pandemic: Findings from an online cross-sectional survey
Source: PLoS One. 2022 Aug 25;17(8):e0272905. doi: 10.1371/journal.pone.0272905 (PMC9409578; doi:10.1371/journal.pone.0272905)
Supplement: Attachment — Submitted filename: Facebook Comment.docx [file pone.0272905.s003.docx]

**Reviewer #1: The research idea and manuscript is indeed interesting and relevant in today’s world**

**Author Responses:** Thanks for your complement for our working.

**The manuscript would benefit grammatical revisions**

**Author Responses:** Thanks for your comment. We revised it.

**If abbreviations are being used, they should be use consistently throughout the paper.**

**Author Responses:** Thanks for your comment. We revised it in line 255.

**Abstract:
In the background- There could be more generalizability to the topic. Rather than using the phrase Bangladeshi university students, is this study generalizable to all university students during the covid-19 pandemic**

**Author Responses:** Thanks for your comment. We revised it in line 4-24.

**Methods
What is Google form?**

**Author Responses:** Thanks for your comment. Due to the COVID-19 pandemic, there were not possible to collect data through a face-to-face interview. We collected data by google form. The Google forms had several advantages which a manual questionnaire does not have, namely paperless, environmentally friendly, time-efficient, labour costs, accurate recapitulation of respondents' answers, and practical. Line 96-100.

**COVID-19 related information questionnaire- what is the origin of these questions? Are they derived from any validated survey or the authors self-validated it by running a pilot?**

**Author Responses:** Thanks for your comment. COVID-19-related information consists of twelve questions. We run a pilot study for these 12 questions before rolling it out for this study. Both reliability and sensitivity were good. The Cronbach alpha for these questions was 0. 864. Line 132-134.

**How is this information different from other studies performed about Facebook addiction?**

**Author Responses:** Thanks for your comment.

In the first half of the COVID-19 pandemic, there were some studies regarding Facebook addiction in Bangladesh, but there is no study who find out the association of Facebook addiction and COVID-19 related information in Bangladesh. Many studies suggested that Facebook addiction is increased for COVID-19 related information(<https://doi.org/10.3389/fpsyg.2021.682837>). For that, we included these questions. This was one of the unique for our study. Also, this study will help to trend of Facebook addiction in Bangladesh.

**Your results are quite similar to studies that were performed in pre covid-19 pandemic era. So, how would you factor in the COVID-19 pandemic and its resultant isolation in your study.**

**Author Responses:** Thanks for your comment.

There were a few studies conducted in Bangladeshi University in pre pandemic. To conduct the actual scenery more research had to conduct. Although two studies in pre pandemic in Jahangirnagar University shown Facebook addiction can be addiction was 39.7%-78.8% and both studies were completed in 2018 with 300 sample sizes. (<https://www.neliti.com/publications/263131/impact-of-facebook-obsession-among-university-students-in-bangladesh>, DOI: [10.1016/j.psychres.2018.12.039](https://doi.org/10.1016/j.psychres.2018.12.039), <https://doi.org/10.1016/j.jval.2018.04.1284>). That mean there were huge variation of the percentages. Many research showed peoples fear and psychological subject were prone to decline trend after the 1^st^ wave of Covid-19( <http://dx.doi.org/10.1136/bmjopen-2020-040620>, <https://doi.org/10.2147/PRBM.S354083>) . Many people are starting to ignoring the national guideline (<https://www.aa.com.tr/en/asia-pacific/2nd-wave-of-covid-19-hits-bangladesh/1983091>) of COVID-19 after 1^st^ wave. Our study conducted in the mid-2021 of COVID-19 pandemic in Bangladesh. Although COVID-19 information is linked to Facebook addiction(<https://doi.org/10.3389/fpsyg.2021.682837>). That can affect our result.

**The result that you have reported that physical activity is associated with increased Facebook addiction is different from previous studies. But no thought process is there to try to explain the difference. Could it be, for example that while being on an exercise machine people use social media to divert attention?**

**Author Responses:** Thanks for your comment.

This study revealed that those involved in any physical activities in this pandemic had higher Facebook addiction. A prior study also showed that problematic smartphone use is linked to physical inactivity during this COVID-19 outbreak [37] and that these trends in smartphone use may increase [37]/decrease [38] the use of Facebook, which turns into an addiction. Other studies also revealed that the increase in physical activities reduces/increase Facebook addiction. [14] [31] [38]. As an adaptation measure to personal and work life, COVID-19-induced lockdown resulted in people becoming more attached to their smartphones [32] [33], and such problematic use may lead them to use their smartphones and social media sites even during any type of physical activity like a workout. But the inconsistency between these two findings varies due to unclear reasons. This inconsistency may occur for the “Internet Plus Exercise” campaign to promote health.

**I have made comments to highlighted areas in a pdf copy of the manuscript as well**

**Reviewer #2: Nice manuscript. The authors want to determine the factors associated with FA among university students.**

**Author Responses:** Thanks for your compliment.

**English writing needs some improvement.**

**Author Responses:** Thanks for your comment. We revised it.

**Discuss only the results/ the concept should be in the introduction section.**

**Author Responses:** Thanks for your comment. We revised it.

**Following the author's recommendations (table and results). Results: all Tables need some editing.**

**Author Responses:** Thanks for your comment. We revised it.

**References: typo in accord with the guideline’s authors**

**Author Responses:** Thanks for your comment. We revised it.
