# Supplementary material for: Factors associated with Facebook addiction among university students amid the COVID-19 pandemic: Findings from an online cross-sectional survey
Source: PLoS One. 2022 Aug 25;17(8):e0272905. doi: 10.1371/journal.pone.0272905 (PMC9409578; doi:10.1371/journal.pone.0272905)
Supplement: Attachment — Submitted filename: PLOS ONE.docx [file pone.0272905.s004.docx]

Editor Comments:

Please detail survey pilot testing and validation using standard reporting guidelines. Rest of the revision seems adequate

**Author responses:**

COVID-19-related information consists of twelve questions. We run a pilot study for these 12 questions before rolling it out for this study. Before the initiation of data collection, we conducted a pilot study to determine whether the study's questionnaires were understandable for the general public, particularly for university students, and to examine the viability of the data collection tools for gathering data swiftly without imposing onerous conditions. Upon completing the pilot study, we revised some questions and altered wordings as per the convenience of the respondents. In addition, the pilot study was also conducted to determine whether the data showed too much or too little variability and to cross-check the eligibility criteria for the respondents. To assure validity, the researchers committed to upholding the study's credibility at every stage of the data collection and analysis process. We further reinforced the credibility by ensuring that each respondent understood the questionnaire and the purpose of the study. Both reliability and sensitivity were good. The Cronbach alpha for these questions was 0.864.
